# Contextualizing the revised Patient Perception of Patient-Centeredness (PPPC-R) scale in primary healthcare settings: a validity and reliability evaluation study

**DOI:** 10.1186/s12875-023-02227-x

**Published:** 2024-01-05

**Authors:** Yiyuan Cai, Pengfei Guo, Jiong Tu, Mengyao Hu, Lingrui Liu, Bridget L. Ryan, Jing Liao, Rubee Dev, Yiran Li, Tianyu Huang, Ruilin Wang, Li Kuang, Ruonan Huang, Xinfang Li, Edmundo Roberto Melipillán, Shuaixiang Zhao, Wenjun He, Xiaohui Wang, Nan Zhang, Dong (Roman) Xu

**Affiliations:** 1 Department of Epidemiology and Health Statistics, School of Public Hhealth, Guizhou Medical University, Gui’an, China; 2 Department of Environmental Health Sciences, Yale School of Public Health, Yale University, New Haven, USA; 3 School of Sociology and Anthropology, Sun Yat-Sen University, Guangzhou, China; 4 Survey Research Center, University of Michigan, Ann Arbor, MI USA; 5 Department of Health Policy and Management, Yale School of Public Health, Yale Center for Methods in Implementation and Prevention Science, New Haven, USA; 6 Departments of Family Medicine and Epidemiology and Biostatistics, Western University, London, ON Canada; 7 Department of Health Statistics, School of Public Health, Sun Yat-Sen University, Guangzhou, China; 8 Faculty of Applied ScienceSchool of Nursing, University of British Columbia, Vancouver, Canada; 9 Interdisciplinary Center Psychopathology and Emotion Regulation, Department of Psychiatry, University Medical Center Groningen, University of Groningen, Groningen, Netherlands; 10 School of Sociology and Population Studies, Renmin University of China, Beijing, China; 11 Department of Health ManagementSchool of Public Health, Sun Yat-Sen University, Guangzhou, China; 12 The First Affiliated Hospital of Xi’an Jiao Tong University, Xi’an, China; 13 13Dong Fureng Institute of Economic and Social Development, Wuhan University, Wuhan, China; 14 Facultad de Psicología, Universidad del Desarrollo, Sede Santiago, Chile; 15 Jiaozuo People’s Hospital, Jiaozuo, China; 16 Acacia Lab for Implementation Science, School of Health Management and Dermatology Hospital, Southern Medical University, Guangzhou, China; 17 Department of Social Medicine and Health ManagementSchool of Public Health, Lanzhou University, Lanzhou, China; 18 School of Health Management, Inner Mongolian Medical University, Hohhot, China; 19 Center for World Health Organization Studies and Department of Health Management, School of Health Management, Southern Medical University, Guangzhou, China; 20 Southern Medical University Institute for Global Health (SIGHT), Dermatology Hospital of Southern Medical University (SMU), Guangzhou, China

**Keywords:** Patients-Centeredness Care, Quality of health care, Factor analysis, Localization and Validation

## Abstract

**Background:**

An English version of the Patient Perception of Patient-Centeredness (PPPC) scale was recently revised, and it is necessary to test this instrument in different primary care populations.

**Aim:**

This study aimed to assess the validity and reliability of a Chinese version of the PPPC scale.

**Design:**

A mixed method was used in this study. The Delphi method was used to collect qualitative and quantitative data to address the content validity of the PPPC scale by calculating the Content Validity Index, Content Validity Ratio, the adjusted Kappa, and the Item Impact Score. Confirmatory factor analysis (CFA) and exploratory factor analysis (EFA) were used to assess the construct validity of the PPPC scale through a cross-sectional survey. The internal consistency was also assessed.

**Setting/participants:**

In the Delphi consultation, seven experts were consulted through a questionnaire sent by email. The cross-sectional survey interviewed 188 outpatients in Guangzhou city and 108 outpatients in Hohhot City from community health service centers or stations face-to-face.

**Results:**

The 21 items in the scale were relevant to their component. The Item-level Content Validity Index for each item was higher than 0.79, and the average Scale-level content validity index was 0.97 in each evaluation round. The initial proposed 4-factor CFA model did not fit adequately. Still, we found a 3-factor solution based on our EFA model and the validation via the CFA model (model fit: ${\chi }^{2}=294.573$, *P* < 0.001, RMSEA = 0.044, CFI = 0.981; factor loadings: 0.553 to 0.888). Cronbach's α also indicated good internal consistency reliability: The overall Cronbach's α was 0.922, and the Cronbach's α for each factor was 0.851, 0.872, and 0.717, respectively.

**Conclusions:**

The Chinese version of the PPPC scale provides a valuable tool for evaluating patient-centered medical service quality.

**Supplementary Information:**

The online version contains supplementary material available at 10.1186/s12875-023-02227-x.

## What is already known about the topic?

Using the patient-centered clinical method conceptual framework to assess the quality of the patient-clinician relationship may improve patient-centered care quality in communication and shared decision-making.

However, no valid patient-centered care scale is yet available for use in primary healthcare settings in China.

## What this paper adds

The Chinese version of the PPPC scale demonstrated rigorous validity and reliability of three distinct factors and covered the four components under the patient-centered clinical method conceptual framework.

## Implications for practice, theory, or policy

The Chinese version of the Patient Perception of Patient-Centeredness scale is valuable for evaluating patient-centered medical service quality.

This leading study bridges the evidence gap in measuring patient-centered care quality in China.

## Introduction

Patient-centered care is one of the components of the quality of care defined by the Institution of Medicine (IOM) [1]. The quality of patient-centered care improvement has been associated with various positive health system outcomes, such as effectively enhanced patient satisfaction, improved health-related outcomes(e.g., improvement of symptoms), reduced avoidable referrals, and diagnostic costs [2–6]. If the provider bridges the gap between clinical quality and patient perception, it could improve medical utilization and clinical quality [7]. Mohammed's systematic review found that patients can perceive ten domains of patient-centered care quality (communication, access, shared decision-making, provider knowledge and skills, physical environment, patient education, electronic medical record, pain control, discharge process, and preventive services) [8]. When doctors provide better doctor-patient communications, patients would experience more patient-centered care from doctors, and the reciprocal trust between doctors and patients may improve the effectiveness of treatment plans and, ultimately, clinical outcomes [9]. Therefore, the evaluation of patient-centered care quality can be constructed to focus on communication, accessibility, and quality [10].

The Patient-Centered Clinical Method (PCCM) [11] is most frequently cited in family medicine, [2] which has been used as a guide for practitioners working to improve the quality of patient-centered care that they deliver to patients through enhanced communication skills [9, 12]. Little et al.’s study showed that over 77% (599 out of 781 consecutive patients in the waiting room of three doctors’ surgeries) of patients endorsed and had expectations of care that were the elements of patient-centered care [11, 13]. According to the PCCM, patient-centered concepts incorporate four interactive components used in the setting of primary care [11]: 1) exploring health, disease, and the illness experience, 2) finding common ground, 3) understanding the whole person, and 4) enhancing the patient-clinician relationship. These four components also encompassed patient-clinician relationship aspect quality: communication and shared design making [8]. Hence, existing literature acknowledged that using the PCCM conceptual framework to assess the quality of the patient-clinician relationship may improve patient-centered care quality in communication and shared decision-making. However, in low- and middle-income countries, due to limited time and human resources, [14] providers are more likely to focus on improving clinical and health system quality to enhance patient-centered care quality rather than pay attention to patient perspective.

Currently, no valid patient-centered care scale is available for use in primary healthcare settings in low- and middle-income countries [2, 12]. Developed based on the PCCM conceptual framework [11], the Patient Perception of Patient-Centeredness (PPPC) scale was widely used in many countries in different patient groups and healthcare settings to assess patient-centered care quality [4–6, 9, 15–20]. Most of the patient-centered care scales reflected only two or three of the four commonly used domains [2]. In the latest version, Ryan et al. revised the PPPC scale to reflect the four widely used domains of the clinicians' interactive activities in primary care settings. This revised PPPC scale was used to assess patient-centered care characteristics and a practical tool for providers to improve their interactions with patients for patient-centered care practice improvements [9]. In this study, we chose a 21-item PPPC scale which contained 18 items from the Ryan et al. version and three items from the original 14-item version [5] that were not included in the revised version to conduct the content validity and construct validity assessment to reflect the quality of patient-centered care in public health care settings more comprehensively.

## Methods

### Study design and procedures

**Fig. 1:**
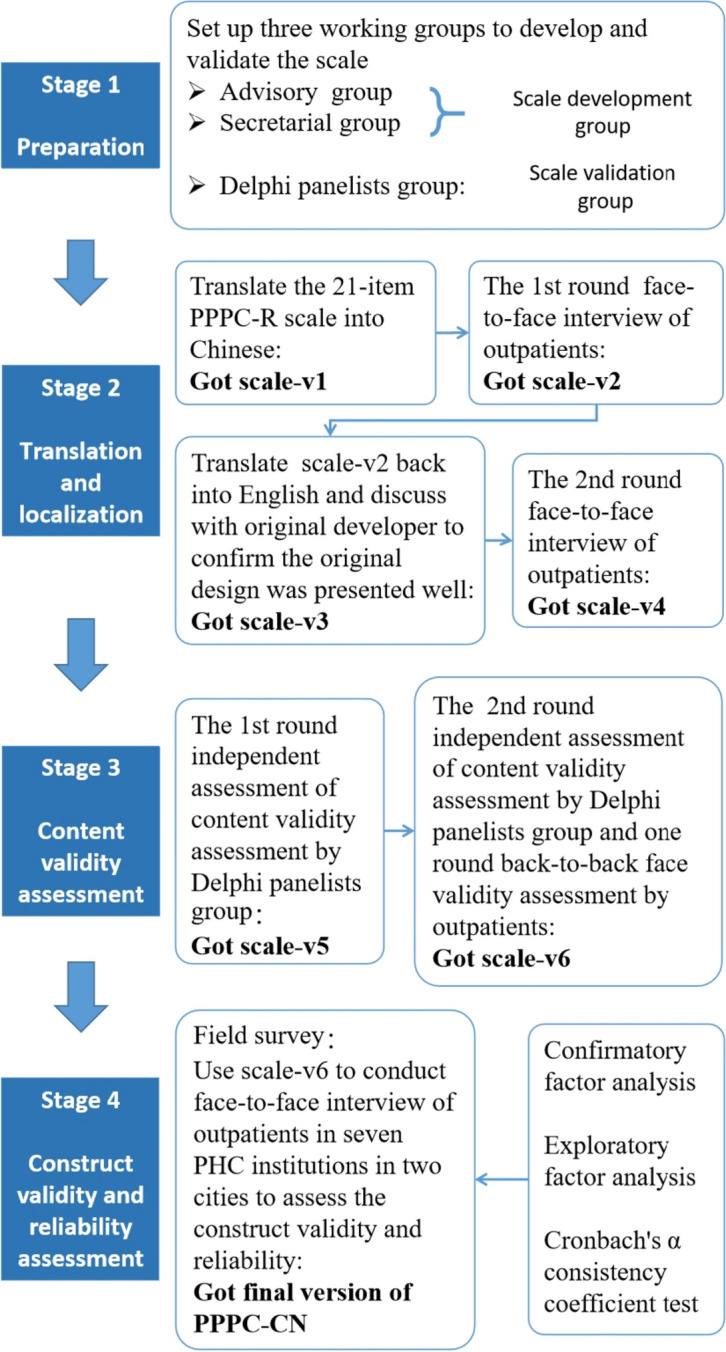
Procedures of PPPC-CN scale localization and validation

This study was separated into four stages (Fig. 1) and used a mixed method. In stage one, three working groups were set up to prepare the scale localization and validation processes. In stage two, the scale development group translated the 21-item PPPC-R scale into Chinese and conducted two rounds of face-to-face outpatient interviews to complete the face validity assessment. The outpatients were invited directly by asking for their participation willingness in the outpatient waiting room. We also conducted a round translate back process to refine the scale. In the third stage, we invited seven experts from different disciplines, including one medical psychologist, one general practitioner, one public health physician, two epidemiologists, and two health management panelists, who evaluated the scale's content validity through online questionnaires sent by email separately. The researchers invited these experts through their work net. In the fourth stage, we followed the cross-cultural adaptation process [21, 22] and the COnsensus-based Standards for the selection of health Measurement INstrument (COSMIN) guideline [23] to conduct our study. The detailed scale localization validation procedures were presented in Additional file 1: Appendix 1, including both qualitative and quantitative approaches.

### Settings, sampling, and investigate processes

For construct validity evaluation and internal consistency reliability, the sample size needs to be 10 times the number of items. Therefore, at least 210 respondents were needed [24]. We estimated a 90% response rate to account for potential non-response. Therefore, 223 respondents were needed. By using convenience sampling in urban and suburban regions in Guangzhou and Hohhot cities, seven community health service centers were selected (institutions’ list presented in Additional file 1: Appendix 2). From July 20 to 26, 2019, the outpatients visited the internal medical physicians or general practitioners in selected community health service centers, and those aged≥18 years were eligible. Anyone with hearing or language impairment was excluded. When the participants left the consultation room, our investigator asked about their willingness to participate in our survey. If they were willing to participate after our investigator informed the content of the informed consent, they would be enrolled in our study. After they finished the survey, we provided them with a towel as a gift(cost RMB 5 yuan) and thanked their participation. Our investigators used a REDCap [25] based online form to complete the one-to-one, face-to-face survey.

### Statistical analysis

The Content Validity Index (I-CVI), Content Validity Ratio (CVR), the adjusted Kappa, and the Item Impact Score (IIS) were used to assess the content validity [26]. Because the item in PPPC-R was designed under the PCCM conceptual framework [5], we used confirmatory factor analysis (CFA) to test whether the proposed four domains, which were 1) exploring health, disease, and the illness experience, 2) finding common ground, 3) understanding the whole person, and 4) enhancing the patient-clinician relationship could be imposed as a 4-factor CFA model by using our sample. Suppose this structure does not fit the data well. In that case, we would then use the exploratory factor analysis (EFA) to 1) identify the number of factors and 2) determine which item corresponds to each factor. The CFA model and EFA model were tested using Mplus Version 7.0 with the weighted least squares means and variance (WLSMV) estimator to account for the categorical, ordinal nature of the items and the fact that distributions of the item responses were skewed [27]. The number of factors was determined by the characteristic roots (≥ 1) or the cumulative explained variance (≥ 50%). The scree plot was also used as a guide to choose the number of factors. A threshold factor loading greater than 0.30 was used to decide to accept an item as belonging to a factor [28]. If one item was loaded into two factors in the EFA model, the one with a higher loading value would be chosen [27]. The following model fit indices and their criteria were used to determine the best model [24, 29]: -comparative fit index (CFI) greater than 0.90; α the Turker Lewis Index (TLI) greater than 0.90; β approximate error root mean square (RMSEA) and its 90% confidence interval, RMSEA less than 0.06 indicates good; χchi-square/degree of freedom (*χ*^*2*^*/df*) less than 3.0. The Cronbach's α consistency coefficient was calculated to determine the scale's internal consistency as a whole and each factor, respectively [30, 31]. For the whole scale and sub-domain of the scale, we calculate Cronbach's *α.* When a value *of* more than 0.7 was acceptable [27]. Response patterns were also presented, which were the proportion of each item's different choices. The item has better discrimination when the item has endorsement rates between 0.2 and 0.8 [27].

Descriptive statistics were performed by SPSS 22.0 to assess the general participant characteristics. Means and variances were adopted to describe normal distribution data. Medians and quartiles were used to describe non-normally distributed data. The group difference was tested using the t-test or ANOVA for normally distributed data, and the Wilcoxon rank-sum test or Kruskal–Wallis test for non-normally distributed data; *P* ≤ 0.05 indicates statistical significance.

## Results

### Adaptation and content validation

In stage 2, after translation, researchers launched two rounds of interviews to contextualize the PPPC-CN scale. Seven outpatients were willing to accept face-to-face interviews to complete the scale-v1 or scale-v3 and provide their opinions of their understanding of each item at public healthcare institutions in Guangzhou and Hohhot City. The main changes made on the scale-v4 were based on feedback from eight outpatients in the second round of interviews, and the researcher revised the scale consistently and achieved the initial localization of the scale, that is, the item expressing and connotation in Mandarin matched local health system and service and context. Revision details are presented in Additional file 1: Appendix 3.

Seven experts and seven outpatients participated in the content validation and face validation consultations. The results of the offline independent assessment of the validation of the scale by Delphi panelists and target users (outpatients) in stage 3 are presented in Table 1. In both rounds of content validity assessment, the I-CVI for each item was more than 0.79, and the S-CVI/AVE was 0.97 in each evaluation round. The results indicated that the scale items were relevant to the factor they belong to, and the scale was relevant to the measurement purpose. However, during the first round of evaluation, three items were unclear, and 12 items were rated as "useful, but not essential" by one to three experts, with CVR scores below 0.99 for these items, suggesting that they should be eliminated. In the second evaluation round, two items were considered unclear, and nine out of the 12 items mentioned above recommended to eliminate were considered "essential". Only three items were rated as "useful but essential" by one expert. However, in the second evaluation round, there was one "essential" item identified as "useful but not essential" by one expert and three items rated as "useful but not essential" by two experts. At the same time, seven patients who had seen a doctor in the CHC clinic in the previous month were willing to participate in the scale’s face validity evaluation. The evaluation results showed that two items' IIS < 1.5, suggesting that they were unimportant and should be eliminated. Based on these comments, we added some minor revisions to the expression of the item and then got the scale-v6. However, we did not eliminate any item assessed as "useful but not essential" or items with IIS < 1.5. After each round of evaluation, we refined the items' phrasing without modifying the components to which the items belonged. We improved the efficiency by conducting translation and content validity analysis simultaneously. Finally, before conducting field surveys, all items in the scale-v6 were assessed under appropriate components and items.

**Table 1:** Calculation of I-CVI, Clarity, and CVR for 21-item PPPC-CN items (*n*=7)

Item	Round 1 assessment based on scale-v3						Round 2 assessment based on scale-v4						Face validity
	N_i_^a^	I-CVI^b^	K of I-CVI^c^	N_c_^d^	N_e_^e^	CVR^f^	N_i_^a^	I-CVI^b^	K of I-CVI^c^	N_c_^d^	N_e_^e^	CVR^f^	IIS ^g^
1	7	1.00	1.00	3	7	1.00	7	1.00	1.00	7	7	1.00	2.00
2	6	0.86	0.85	5	6	0.71	7	1.00	1.00	6	7	1.00	3.00
3	6	0.86	0.85	6	7	1.00	7	1.00	1.00	6	5	0.43	2.80
4	7	1.00	1.00	5	6	0.71	7	1.00	1.00	6	7	1.00	3.00
5	7	1.00	1.00	6	5	0.43	7	1.00	1.00	6	7	1.00	2.80
6	7	1.00	1.00	6	7	1.00	7	1.00	1.00	6	7	1.00	3.60
7	7	1.00	1.00	7	6	0.71	7	1.00	1.00	7	7	1.00	1.50
8	7	1.00	1.00	7	6	0.71	7	1.00	1.00	6	7	1.00	1.00
9	7	1.00	1.00	5	7	1.00	7	1.00	1.00	5	6	0.71	3.10
10	7	1.00	1.00	5	7	1.00	7	1.00	1.00	6	7	1.00	2.90
11	7	1.00	1.00	6	6	0.71	7	1.00	1.00	7	7	1.00	3.70
12	7	1.00	1.00	2	5	0.43	7	1.00	1.00	6	7	1.00	2.20
13	6	0.86	0.85	4	4	0.14	7	1.00	1.00	6	7	1.00	2.70
14	7	1.00	1.00	5	7	1.00	6	0.86	0.85	6	6	0.71	2.70
15	7	1.00	1.00	7	5	0.43	7	1.00	1.00	7	6	0.71	1.90
16	6	0.86	0.85	4	6	0.71	7	1.00	1.00	7	6	0.71	2.00
17	7	1.00	1.00	6	3	-0.14	6	0.86	0.85	5	6	0.71	1.50
18	7	1.00	1.00	5	7	1.00	6	0.86	0.85	7	7	1.00	2.00
19	7	1.00	1.00	6	7	1.00	6	0.86	0.85	6	6	0.71	1.40
20	7	1.00	1.00	6	7	1.00	6	0.86	0.85	6	7	1.00	2.10
21	6	0.86	0.85	6	6	0.71	7	1.00	1.00	7	7	1.00	2.80

*Abbreviations*: *I-CVI* Content Validity Index, *CVR* Content Validity Ratio, *IIS* Item Impact Score

^a^N_i_, the number of panelists who rated 3 or 4 out of 4 points, supporting the item's relevance

^b^Interpretation of I-CVIs: The item would be appropriate if the I-CVI is higher than 79 percent. If it is between 70 and 79 percent, it needs revision. If it is less than 70 percent, it should be eliminated

^c^Adjusted Kappa, K=(I-CVI-P_c_)/(1-P_c_) [P_c_ (probability of a chance occurrence) was computed using the formula: P_c_=[N!/A!(N-A)!]×0.5^N^, where *N*=number of experts=7, and A=number of panelists who agreed that the item is relevant=N_i_.] Interpretation of K of I-CVI, values above 0.74, between 0.60 and 0.74, and between 0.40 and 0.59 are considered as excellent, good, and fair, respectively

^d^N_c_, the number of panelists who rated 3 or 4 out of 4 points, supporting the clarity of the expression of the item

^e^Interpretation of item impact score. The item would be eliminated when the score is less than 1.5

^f^N_e_, the number of panelists who rated 3 out of 3 points, supporting the item's necessity

fInterpretation of CVR: if CVR is greater than 0.99, the item would remain at the instrument, and the rest should be eliminated

^g^IIS=frequency (the percent of patients who scored 4 or 5 out of 5 on the item importance)×importance (mean important score of the item). If the IIS of the item≥1.5, it would be maintained in the instrument; otherwise, it should be eliminated

### Construct validity assessment

#### Characteristics of study participants

Our study used the scale-v6 to complete the field survey in stage 4. There were 296 (63.4%, 467 were invited in total) outpatients who completed one-to-one, face-to-face interviews using the 21-item adapted scale (scale-v6), 188 in Guangzhou city and 108 in Hohhot city. The information on the institution is presented in Additional file 1: Appendix 2). There was no statistical difference in mean age, gender, and the proportion of education level between the two cities' participants (Additional file 1: Appendix 4). Among the study participants, most outpatient individuals in Guangzhou visited clinicians for follow-up consultations, and nearly 70% of outpatients visited general practitioners to complete their consultations. The two main reasons for their clinical visits were getting a common cold and seeking care for prescribed medication for hypertension treatment.

#### Factor analysis

**Fig. 2:**
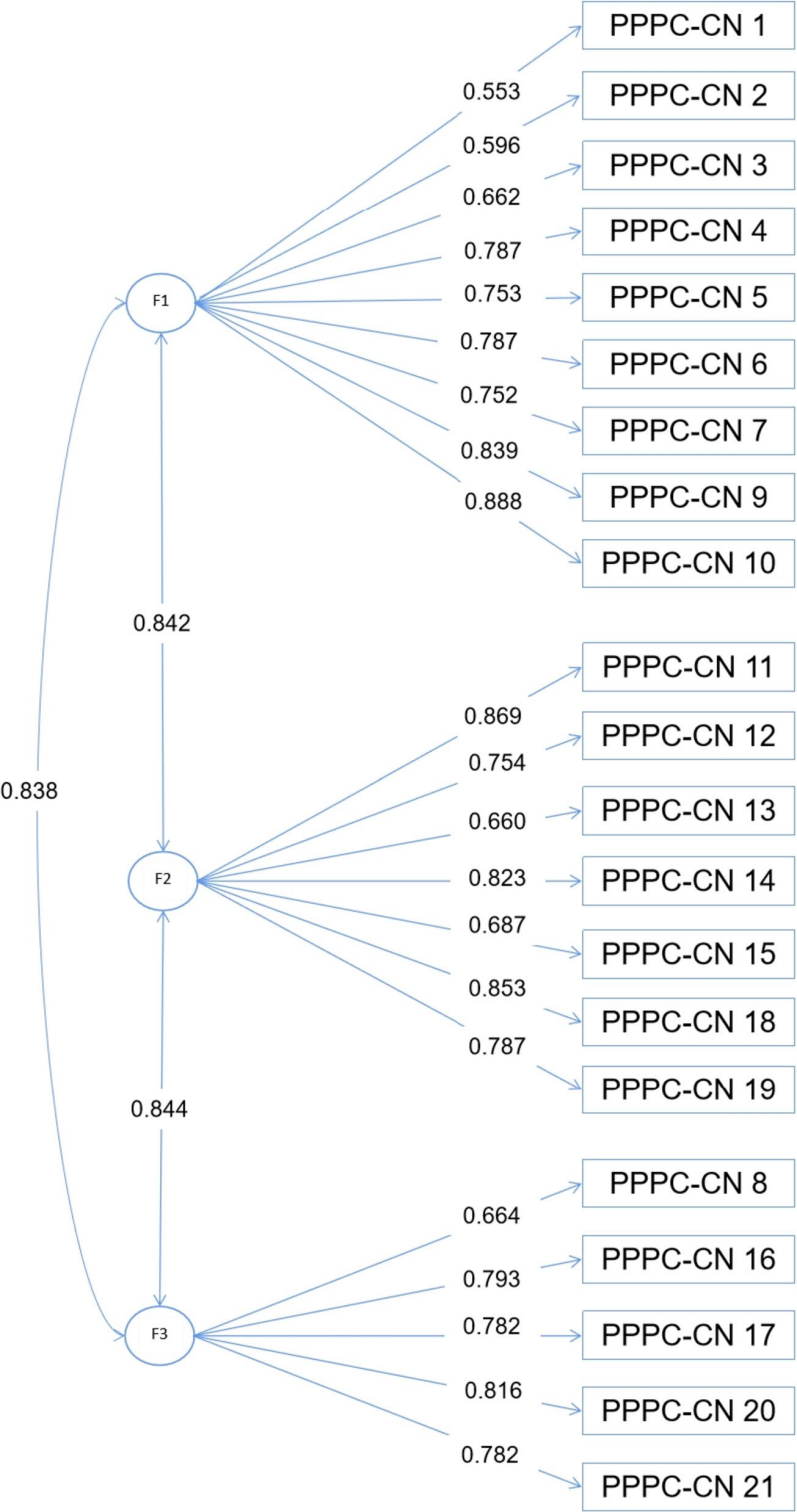
The final CFA result of a 3-factor structure for the 21-item Chinese version PPPC-R scale (standardized parameter estimates, *P*<0.001)

Our initial CFA model used data from 296 outpatients. However, the initial constrained 4-factor CFA model did not fit well [27]: ${\chi }^{2}=455.61$, *P* < 0.001, ${\chi }^{2}/df=2.435$, RMSEA = 0.072 (90%CI: 0.064 ~ 0.081), CFI = 0.906, TLI = 0.891. Though the CFI was greater than 0.90, the TFI was less than 0.90, suggesting that the model did not fit well. The correlations and loadings used to define factors were statistically significant (*P* < 0.01). Then, we used the same data for the EFA. Bartlett's spherical test result presented KMO = 0.931. Three factors were extracted according to the characteristic roots, greater than 1 in the scree plot, and the cumulative explained variance is 54.95% (Additional file 1: Appendix 5). All 21 items were allocated into three factors. Correlations among factors were 0.453 for F1 with F2, 0.558 for F1 with F3, and 0.510 for F2 with F3. Correlations and loadings used to define factors are statistically significant (*P* < 0.05). The factor loadings range from 0.337 to 0.874. The loading for the item is shown in Table 2. This 3-factor solution was conceptually reasonable based on this EFA model and had a good model fit with ${\chi }^{2}=192.756$, *P* = 0.011, ${\chi }^{2}/df=1.283$, RMSEA = 0.031 (90%CI:0.016 ~ 0.043), CFI = 0.993, TLI = 0.990. To confirm whether the final EFA model fits well, we use the CFA to validate it. The CFA model also had a good model fit with ${\chi }^{2}=294.573$, *P* < 0.001, ${\chi }^{2}/df=1.584$, RMSEA = 0.044 (90%CI: 0.035 ~ 0.054), CFI = 0.981, TLI = 0.979. Figure 2 presents the relatively high loadings, ranging from 0.553 to 0.888. Correlations among the factors ranged from 0.838 to 0.844 (Fig. 2). Table 3 presents the commonality of items belonging to the components in the original scale and our new 3-factor scale.

**Table 2:**
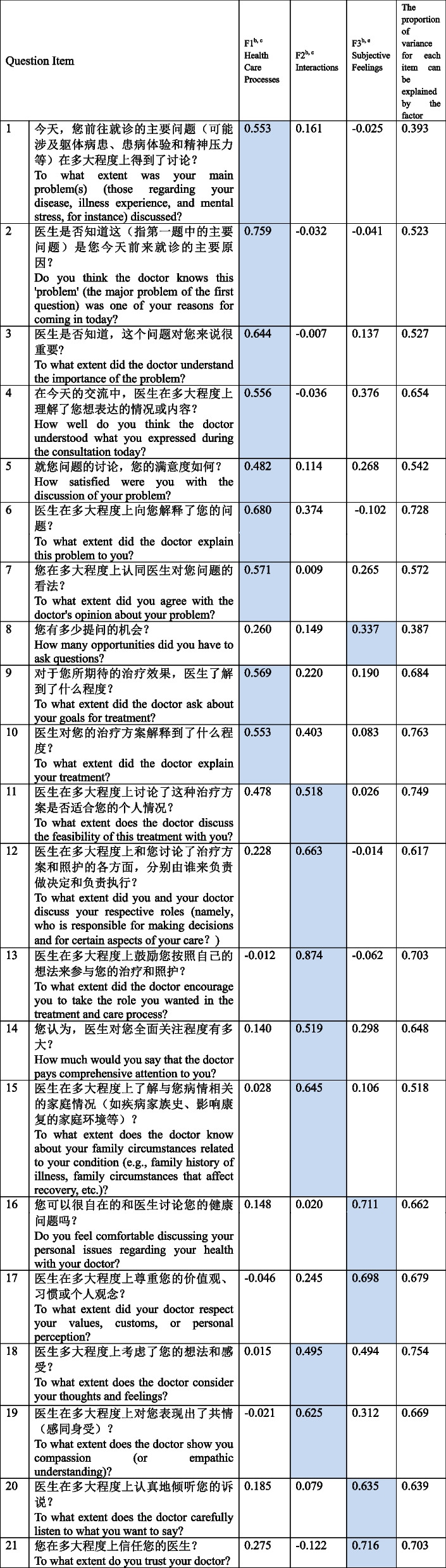
Factor loadings in EFA of the PPPC-CN ^a^ scale

^*^The blue highlighted boxes indicate these items loaded in the same factor in the same column

^a^PPPC-CN, Chinese version of revised Patient Perception of Patient-Centeredness scale;

^b^Factor loadings (i.e., pattern coefficients) with Geomin rotation

^c^F1 can be defined as "Health Care Processes"

^d^F2 can be defined as "Interactions between patient and doctor."

^e^F3 can be defined as "Subjective Feelings of the patient after consultation."

**Table 3:** The number of PPPC-CN items loading onto each factor by the factors of the PCCM conceptual framework

PCCM Conceptual Framework	F1: Health Care Process	F2: Interaction	F3: Subjective Feeling of Patient-Centered Care
Component 1: exploring health, disease, and the illness experience	4	1	0
Component 2: understanding the whole person	0	2	2
Component 3: finding common ground	5	3	1
Component 4: enhancing the patient-clinician relationship	0	1	2
Total number of items in each factor	9	7	5

#### Response patterns and reliability

Table 4 presents the 21-item means and standard deviations and response patterns. All items present high endorsement rates in alternatives 1 and 2. No option has an endorsement proportion over 0.95. However, alternative 4 has the lowest endorsement proportion (lower than 0.05). The overall Cronbach's α value for the 21 items was 0.922, and the Cronbach's α value for each factor was 0.851, 0.872, and 0.717, respectively.

**Table 4:** Descriptive statistic of 21-item PPPC-CN items by the factor (Cronbach's α)

No. of item	Items and Alternatives (21-item Cronbach α: 0.992)	n	Mean	SD	Response Category and Proportion	
F1 (Cronbach α: 0.851)						
1	To what extent was your main problem(s) (those regarding your disease, illness experience, and mental stress) discussed? 1. Completely 2. Mostly 3. A Little 4. Not at all	296	1.63	0.74	1	0.493
					2	0.416
					3	0.057
					4	0.034
2	Do you think the doctor knows this 'problem' (the major problem of the first question) was one of your reasons for coming in today? 1. Yes 2. Possible 3. Uncertain 4. No	296	1.25	0.59	1	0.814
					2	0.135
					3	0.037
					4	0.014
3	To what extent did the doctor understand the importance of the problem? 1. Yes 2. Possible 3. Uncertain 4. No	296	1.23	0.53	1	0.807
					2	0.162
					3	0.020
					4	0.010
4	How well do you think the doctor understood what you expressed during the consultation today? 1. Completely 2. Mostly 3. A Little 4. Not at all	296	1.34	0.55	1	0.686
					2	0.294
					3	0.010
					4	0.010
5	How satisfied were you with the discussion of your problem? 1. Very satisfied 2. Satisfied 3. Somewhat satisfied 4. Not very satisfied	296	1.44	0.56	1	0.591
					2	0.389
					3	0.014
					4	0.007
6	How satisfied were you with the discussion of your problem? 1. Completely 2. Mostly 3. A Little 4. Not at all	296	1.49	0.66	1	0.591
					2	0.348
					3	0.044
					4	0.017
7	To what extent did you agree with the doctor's opinion about your problem? 1. Completely 2. Mostly 3. A Little 4. Not at all	296	1.44	0.58	1	0.605
					2	0.358
					3	0.034
					4	0.003
9	To what extent did the doctor ask about your goals for treatment? 1. Completely 2. Mostly 3. A Little 4. Not at all	296	1.52	0.65	1	0.554
					2	0.389
					3	0.044
					4	0.014
10	To what extent did the doctor explain your treatment? 1. Completely 2. Mostly 3. A Little 4. Not at all	296	1.56	0.68	1	0.530
					2	0.395
					3	0.057
					4	0.017
F2 (Cronbach α: 0.872)						
11	To what extent does the doctor discuss the feasibility of this treatment with you? 1. Completely 2. Mostly 3. A Little 4. Not at all	296	1.60	0.76	1	0.530
					2	0.378
					3	0.054
					4	0.037
12	To what extent did you and your doctor discuss your respective roles (namely, who is responsible for making decisions and for certain aspects of your care?) 1. Completely 2. Mostly 3. A Little 4. Not at all		1.73	0.84	1	0.473
					2	0.378
					3	0.095
					4	0.054
13	To what extent did the doctor encourage you to take the role you wanted in the treatment and care process? 1. Completely 2. Mostly 3. A Little 4. Not at all	296	1.85	0.88	1	0.405
					2	0.402
					3	0.128
					4	0.064
14	How much would you say that the doctor pays comprehensive attention to you? 1. Completely 2. Mostly 3. A Little 4. Not at all	296	1.72	0.76	1	0.453
					2	0.395
					3	0.135
					4	0.017
15	To what extent does the doctor know about your family circumstances related to your condition (e.g., family history of illness, family circumstances that affect recovery, etc.)? 1. Completely 2. Mostly 3. A Little 4. Not at all	296	2.03	1.01	1	0.375
					2	0.334
					3	0.176
					4	0.115
18	To what extent does the doctor consider your thoughts and feelings? 1. Completely 2. Mostly 3. A Little 4. Not at all	296	1.65	0.71	1	0.470
					2	0.432
					3	0.078
					4	0.020
19	To what extent does the doctor show you compassion (or empathic understanding)? 1. Completely 2. Mostly 3. A Little 4. Not at all	296	1.76	0.82	1	0.436
					2	0.412
					3	0.108
					4	0.044
F3 (Cronbach α: 0.717)						
8	How many opportunities did you have to ask questions? 1. Very much 2. A little 3. Only a little 4. Not at all	296	1.49	0.69	1	0.598
					2	0.338
					3	0.041
					4	0.024
16	How comfortable are you when discussing your personal issues regarding your health with your doctor? 1. Completely comfortable 2. Mostly comfortable 3. A Little comfortable 4. Not very comfortable	296	1.39	0.60	1	0.669
					2	0.284
					3	0.041
					4	0.007
17	To what extent did your doctor respect your beliefs, values, and customs? 1. Completely 2. Mostly 3. A Little 4. Not at all	296	1.51	0.66	1	0.564
					2	0.372
					3	0.051
					4	0.014
20	To what extent does the doctor carefully listen to what you want to say? 1. Completely 2. Mostly 3. A Little 4. Not at all	296	1.40	0.58	1	0.649
					2	0.311
					3	0.037
					4	0.003
21	To what extent do you trust your doctor? 1. Completely 2. Mostly 3. A Little 4. Not at all	296	1.34	0.55	1	0.696
					2	0.270
					3	0.030
					4	0.003

## Discussion

### Main findings

In this study, we translated a 21-item PPPC scale developed by Ryan et al. [9] into Chinese and validated it with a study sample of 296 outpatients of Guangzhou and Hohhot city to generate a tool for evaluating the patient-centered care quality in outpatients from primary healthcare settings in China. Although the 21 items did not completely align with the four components under the PCCM conceptual framework, the Chinese version of the PPPC scale demonstrated rigorous validity and reliability of three distinct factors and covered the four PCCM components. This pioneering study addressed a subjective assessment of patient-centered care measures in China [9] and bridged the evidence gap in applying patients’ perceptions of measuring the patient-centered care quality.

The scale-v6 was assessed under appropriate components through adaptation and content validation and could comprehensively reflect an underlying highly related, progressive, and interactive relationship of patient-centered service. That is when the physician is “exploring health, disease, and the illness experience”, if the physician “understands the whole person” (of the patient), they are likely to “find common ground” (with the patient) gradually and eventually “enhance the patient-clinician relationship”, [2, 9] to improve health outcomes of the patient [2, 5, 32]. Although our interviews were conducted inside the medical facilities, interviewees only met interviewers in person during the whole interview process, with no physicians or other medical staff on-site to ensure that interviewees’ responses were not affected.

Our final CFA results showed that the 21 items formed a 3-factor scale (Table 4). Specifically, the physician learned about the disease’s progression through communication with the patient (F1). During the interactive discussion about treatment plans (F2), they gradually learned about the patient’s personal and family situations with compassion, improving the patient's perception of care and respect from the physician and ultimately enhancing the patient-clinician relationship (F3).

### Comparison with previous scales

Compared with the results from Ryan et al., [9] F1 in our results contained most items under the component of “health, disease, and the illness experience”, except item 11, which is item 8 in the 18-item PPPC-R scale. Other items stayed consistent with the results from Ryan et al. Our results also aligned with findings from testing the 18-item scale used by Nguyen et al. [4] in the primarily Francophone areas in Canada. From patients' perspective, components 1 and 2 (exploring health, disease, and the illness experience; understanding the whole person) may be closely related and necessary during appointments with physicians, and learning the causes and development of diseases is a mandatory procedure. The combination of most items in these two components may account for health care processes as a whole. This finding suggests that patients from different geographic settings may have similar experiences with a consultation procedure during medical visits.

Items in F2 and F3 in this study differed from Ryan’s study [9] but were similar to those in Nguyen et al. [4]. In this study, F2 contained the items from the PCCM’s four components. Interviewees may understand a progressive relationship among the seven items beneath F2 as follows: after physicians learn about disease development and causes, they start to discuss treatment plans with patients (items 11, 12, and 13); patients can perceive and evaluate the extent of attention paid and compassion shown by physicians during appointments (items 14, 15, 1, 19). Short appointments may restrict further communication, and physicians would formulate treatment plans based on preliminary basic inquiries [14]. As patient-centered approaches become more popular, more general practitioners in primary care settings have adopted the Reason-Ideas-Concerns-Expectations (RICE) approach for inquiry training [33]. This can be traced back to the exact origin of Ideas-Concerns-Expectations (ICE) [34, 35]. The ICE approach also included exploring disease causes during inquiries to improve patient communication and reinforce mutual trust in the patient-clinician relationship. With improved health literacy among the Chinese population, [36] many patients have gained basic health knowledge. They wish to discuss treatment or care plans with healthcare providers [37]. Physicians need to communicate with patients and understand patient concerns to learn about patient expectations regarding treatment outcomes. Meanwhile, patients can feel physicians' full attention and compassion, encouraging more patient-clinician communications. Therefore, the items under F2 can reflect the extent of patient-clinician interactions during medical visits.

The five items in F3 in this study belong to three components in the PCCM. Item 8 reflected the opportunity for the patient to ask questions. Item 16 reflected the comfort of discussing health problems with the physician. Item 17 evaluated the respect from the physician felt by the patient when the patient is expressing personal opinions. Items 20 and 21 assessed the physician’s listening and the patient’s trust in the physician. These five items together indicated the patient’s subjective feelings during the communication with the physician. When patients have more opportunities to ask questions, and as their perceived empowerment enhances, [38] they can express their attitudes and opinions more freely. Patients will acquire more trust in physicians if they receive attentive listening, leading to improved patient-clinician relationships.

F3 in the study by Ryan et al. reflected the patient’s role in the diagnosis and treatment process. However, neither this study nor Nguyen et al. [4] arrived at the same finding. One possible explanation is that outpatients may not be aware of the need to consult with physicians and participate in the treatment plan decision-making (Additional file 1: Appendix 4). In addition, due to restricted time for inquiries, [14] patients may not be able to express opinions adequately or have an in-depth discussion about treatment plans. As a result, they tend to follow decisions by physicians.

### Recommendations for future scale development

When constructing response options for each item, it is critical to distinguish them from each other to reflect the interviewees’ genuine intentions accurately [27]. We analyzed the response patterns in this study and found that few interviewees chose the lowest ratings (D option, endorsement proportion < 0.05), which was close to the result in Ryan et al. [9]. As patient-centered care becomes more widely recognized in healthcare settings, physicians’ service quality is anticipated to improve, and extremely unsatisfying physician service will become rare [38]. Another possible explanation for the relatively high rating is that patients may have a low expectation of health services from primary care facilities. Although our interviews were conducted inside the medical facilities, interviewees only met interviewers in person during the whole interview process, without physicians or other medical staff, to ensure that interviewees’ responses were not affected. Overall, it is unlikely that the option settings were biased, and we concluded that they were appropriate for this scale.

Also, factors need to be distinguished from each other. If factors are not distinct enough to measure a singular characteristic, Cronbach’s α for assessing internal consistency would sharply decline when the number of items is reduced [27]. Our internal consistency analysis for all three factors suggested a modest decline compared with the overall internal consistency as a whole. However, Cronbach’s α scores for each of the three factors were greater than 0.7. The three factors in this scale can both synthetically and independently reflect the patient-centered care service quality from the perspectives of patients.

## Limitations

Three limitations need to be considered for this study. First, previous studies have suggested that PPPC could be more beneficial for patients with experience with multiple medical diagnoses and treatments than for evaluating the patient experience of a single medical appointment [9]. Although we asked participants why they came to the health facility, whether it was their first visit or follow-up check, we did not collect information about their previous medical consultation experiences and encounters related to other illnesses in that health facility. This may affect the results of factor analysis. Second, the facilities selected for this study were chosen through convenience sampling. Although we intended to include diversity in the study sample, such as the regional economy (i.e., developed or undeveloped), facility location (i.e., urban or suburban), and scale (i.e., community health center or health service station), the sample representativeness remains questionable. Randomized sampling should be considered in future research to improve the generalizability of research findings. Third, we did not split our data into two to conduct the EFA and CFA separately because of the sample size limitation. In our future study, we would like to test the 3-factor model further in larger and more diverse samples. Fourth, the PPPC-CN calculated an overall mean score [9] which presented that the lower the score, the better the patient-centeredness care quality. However, this inverse scoring method may not be intuitive [39]. Future design may consider reassigning the scores to achieve a direct scoring scheme.

## Conclusions

The PPPC-CN scale, developed based on the PCCM framework, provides a valuable instrument tool for evaluating patient-centered medical service quality from patients' perspectives. The PCCM framework has represented a progressive relationship among four interacting components to improve the patient-clinician relationship during medical consultations. Future research needs to examine new items that should be designed to achieve a more robust and separately mapping of the questionnaire to the four components of the PCCM framework or use separate scales to assess each component.

### Supplementary Information

**Additional file 1:** **Appendix 1.** Detailed procedures of this study. **Appendix 2.** Healthcare settings. **Appendix 3.** Scale translation and localization for items that are difficult to understand for interviewees. **Appendix 4.** Characteristics of study participants. **Appendix 5.** Factor analysis to determinant the number of factors.

## Data Availability

This study does not share the original data. If there are any special needs, researchers could send an application to caiyiyuan@gmc.edu.cn.
