# Supplementary material for: Contextualizing the revised Patient Perception of Patient-Centeredness (PPPC-R) scale in primary healthcare settings: a validity and reliability evaluation study
Source: BMC Prim Care. 2024 Jan 5;25:11. doi: 10.1186/s12875-023-02227-x (PMC10768460; doi:10.1186/s12875-023-02227-x)
Supplement: Supplementary file 1 — Additional file 1: Appendix 1. Detailed procedures of this study. Appendix 2. Healthcare settings. Appendix 3. Scale translation and localization for items that are difficult to understand for interviewees. Appendix 4. Characteristics of study participants. Appendix 5. Factor analysis to determinant the number of factors. [file 12875_2023_2227_MOESM1_ESM.docx]

## Appendix 1. Detailed procedures of this study.

### Stage 1：Preparation

There were three working groups established for the study. They were the advisory group, the secretarial group, and the Delphi panelist group. The advisory and secretarial groups worked together and were nominated as scale development groups.

1. Advisory group

The group members consisted of experts who major in health systems, anthropology, epidemiology, and health statistics. Their responsibilities were: 1) to develop the work protocol and timeline; 2) to design the content validity rating scale and the face validity rating scale according to COSMIN guidelines^[1]^; 3) to be responsible for theoretically guiding the secretarial group in the localization and validation of the scale, and 4) to invite experts and target users of the PPPC-CN scale to participate in the evaluation of the scale.

1. Secretarial group

The members included two undergraduate students majoring in sociology and one graduate student majoring in social medicine. Their responsibilities were: 1) to translate the scale; 3) to send the content validity evaluation scale and compile the evaluation comments from panelists and selected target users; 4) to report to the advisory about the evaluation comments and give feedback to the Delphi panelists group on the revision results till the scale was finalized.

1. Delphi panelists group

One medical psychologist, one general practitioner, one public health physician, two epidemiologists, and two health management panelists evaluated the scale's content validity. Seven outpatients selected from our target institutions assessed the face validity of the scale.

### Stage 2：Scale translation and localization

We followed the guideline's recommendation to conduct the localization procedures to translate the scale to achieve cross-cultural adaptation^[2]^. The main steps include the following four steps: 1) translating the scale items and adding descriptions (scale version-one, scale-v1); 2) conducting the first round of face-to-face interviews with outpatients who were target users by using the scale-v1 and collecting their feedback on item expressions, and then revised the scale-v1 based on user feedback to obtain the scale-v2; 3) translated back of the scale-v2 and discussed with the original developer^[3]^ to confirm whether the meaning of the localized scale items matched with the developer's original intention, and then, revised the scale-v2 according to the step 4 discussion to obtain the scale-v3; 4) conducted the second face-to-face users' interview by using the scale-v3, and then, revised again according to the results of the interview to obtain the scale-v4. In conducting the face-to-face interview, interviewees only met interviewers in person during the whole interview process. The physicians or other medical staff did not present to ensure that interviewees’ responses were not affected.

### Stage 3：Content validity assessment

The content validation procedure included two rounds of evaluation of content validity by panelists and one round of face validity assessment by outpatients who were target users of the scale when we conducted the second-round content validity assessment. At each round of evaluation, the scale development group further refined the items' Chinese expression based on the content validity assessment to make it more consistent with the Chinese PHC scenario. At the end of this stage, we got an acceptable localized scale (scale-v6) to conduct the field survey to assess the construct validity and reliability of the scale.

The Delphi panelists group evaluated what extent of the relevance of the scale items to the pre-defined domains, the clarity of their expression, and the necessity of the items in the content validity assessment phase^[4-7]^. Item-level Content Validity Index (I-CVI) presented the item's relevance to the component, and Content Validity Ratio (CVR) showed the item's necessity in the component. The item will be appropriate if the I-CVI is higher than 0.79 ^[6]^. The adjusted Kappa was also calculated to avoid the probability of chance agreement in assessment. If adjusted, Kappa above 0.74, between 0.60 and 0.74, or between 0.40 and 0.59 are considered excellent, good, or fair, respectively^[7]^. The average Scale-level content validity index (S-CVI/Ave) was calculated. If the S-CVI/Ave is equal to or larger than 0.90 indicates that the overall content validity of all the items is good^[8]^. Clarity was evaluated quantitatively according to a 4-point ordinal scale (1=not clear, 2=item need some revision, 3=clear, but need minor revision, 4=very clear). The CVR less than 0.99 indicates the item should be eliminated when the number of raters is equal to or less than six^[5]^. Delphi panelists evaluated a comprehensive evaluation of the whole scale qualitatively in two evaluation rounds. The selected target users assessed the face validity by an indicator of Item Impact Score (IIS), which was calculated according to the evaluation by a 5-point Likert scale (5=very important, 4=important, 3=relatively important, 2=slightly important, and 1-unimportant). The IIS was calculated according to the percent of patients who scored 4 or 5 to item importance (frequency) and the mean importance score of the item (importance): IIS= frequency×Importance^[7]^. If the IIS≥1.5, it is maintained in the instrument; otherwise, it is eliminated.

### Stage 4: Construct validity and reliability assessment

We conduct a field survey to collect data to complete the validation of construct validity and assess the reliability. Since the item was designed under the PCCM conceptual framework^[9]^, we used confirmatory factor analysis (CFA) to test whether the original factor structures could be replicated using our sample. Suppose this structure does not fit the data well. In that case, we will then use the exploratory factor analysis (EFA) to 1) identify the number of factors and 2) which item corresponds to each of these factors.^[10]^

## Appendix 2. Healthcare settings.

1. Jingtai street community health service center, Baiyun district, Guangzhou city
2. Donghuan street community health service center, Panyu District, Guangzhou city
3. Xinhua community health service center, Huadu district, Guangzhou city a
4. Fuxing Community Health Service Station, Renmin Road, Saihan District, Hohhot City
5. Renmin Road Community Health Service Center, Saihan District, Hohhot City
6. East Ulanqab Road Community Health Service Center, Saihan District, Hohhot City
7. Civil Administration Armed Police Committee Community Health Service Station, East Ulanqab Road, Saihan District, Hohhot City

## Appendix 3. Scale translation and localization for items that are difficult to understand for interviewees.

In the process of scale translation and localization, we found that most of the interviewees had a different perception of nine of the localized scale items than the developer's intention, including items 1, 2, 4, 12, 13, 14, 15, 17 and 19. These multifaceted reasons provided the basis for our revision, eventually leading to the scale version used for fieldwork.

Item 1 - “To what extent was your main problem(s) (those regarding your disease, illness experience, and mental stress, for instance) discussed?” Interviewees tended to perceive the “main problem(s)” as the disease itself. The main reason was that interviewees went to the doctor mainly due to manifested symptoms or direct health effects of the disease, but routine disruption in illness experience and accompanying mental stress were largely ignored when it came to “main problem(s)”. To enhance the comprehensiveness of understanding on “main problem(s)” among participants, we added appropriate notes to highlight the potential disease, illness experience, and mental stress problems.

Item 4 - "How well do you think the doctor understood what you expressed during the consultation today?" We found that doctors might understand patients by actively querying patients and further learning the patient’s concerns when the patient questioned or provided more personal information regarding illness if the treatment plan was seemingly infeasible or unexpected to that patient. Therefore, we added an explanation in the revised item; that is, this item focused on the extent of the doctor’s understanding of the patient’s situation during the doctor-patient communication, including main problems, related mental burden, environmental impacts on diseases development, and the patient’s expectation for treatment outcomes.

Item 12 - "To what extent did you and your doctor discuss your respective roles (namely, who is responsible for making decisions and who is responsible for certain aspects of your care?)" According to the interviewees for scale-v1, they hardly considered the role of caregivers in their treatment process. They also thought that the patient should follow the authority of the doctor. In most cases, they did not discuss it with doctors. Rationales and solutions are the same as in Item 13.

Item 13 – “To what extent did the doctor encourage you to take the role you wanted in the treatment and care process?” There existed a misunderstanding on scale-v1 for this item. Most interviewees thought that this item was asking to what extent the doctor encouraged them to follow the treatment plan. Some interviewees said that they had not considered possibilities under items 12 and 13 until they were asked in this interview. It is likely because of the following three reasons. First, the “health care system” is a highly professional field, and patients are not empowered with a patient-centered service. They were not used to finding common ground by actively interacting with doctors. Therefore, patients tend to obey guidance from professional medical workers due to a lack of medical background. Second, when primary care facilities provide medical services for patients with chronic illnesses, the primary care facilities mainly offer medications according to prescriptions obtained from hospitals of higher classes. Even when the physician actively queries the patient on illness changes, the patient does not intend to discuss current treatment plans in-depth, probably due to low trust in primary care service quality. Third, the primary care facilities in China usually deal with common diseases such as common colds, diarrheal, and mild fever. Those with common diseases usually go to the doctor for a sense of comfort rather than seek real progress through discussion of treatment plans. Thus, for items 12-13, we explained to the confused interviewees that doctors encourage patients to tackle the task of treatment and care as they wish, splitting up among themselves, their families, and doctors during treatment and recovery.

Item 14 - "How much would you say that the doctor pays comprehensive attention to you?" In pilot interviews, we found that interviewees had a different understanding of the original expression of item 14 than the developer – “How much would you say that the doctor cares about you as a person.” To help the interviewees learn “understanding the whole person”, we translated it as “How much would you say that the doctor pays comprehensive attention to you?” Interviewees found it challenging to understand what “as a person” means and for doctors to pay attention to anything other than diseases. Doctors should consider patients’ social characteristics and environmental impacts on disease development and treatment efficacy. Thus, this current expression reflected the core of this item – “How much would you say that the doctor pays comprehensive attention to you?”

Item 15 - "To what extent did your doctor know about your family (e.g., family history of illness, family environment affecting recovery, etc.)?" We significantly revised this item based on a free translation rather than a literal translation. The original text was “To what extent does your provider know about your family life?” In the initial version, we remained “family life.” But some interviewees thought it “unnecessary” for doctors to learn about their family life, which is too private to tell. This revision was also challenged in expert reviews for the privacy issue and deviation from the inquiry purpose. However, experts agreed that patients should learn about potential impacts on family life if infectious diseases are involved. Therefore, we replaced “family life” with “family”, which is understood as a “family situation” in the Chinese context. In addition, we further added a few examples such as “family history of illness, family environment affecting recovery” to condition on family situations.

Item 17 - "To what extent did your doctor respect your values, customs, or personal perception?" The belief and value in this question may not get demonstrated apparently, because the percentage of the religious population is lower than that in western countries. Therefore, we replaced “belief” with “personal perception” in Chinese. Experts and interviewed patients agreed that religious belief is a less-discussed issue in the doctor-patient conversation in China. But “personal perception” deserves attention. To note, we changed “and” into “or”. As long as the doctor shows respect to any one aspect of value, habit, or personal perception, it is sufficient to reflect the doctor’s attention to the “whole person” of the patient.

Item 19 - "To what extent does the doctor show you compassion (or empathic understanding)?" In pilot interviews, interviewees expressed concerns about understanding “compassion” for less educated people. To help interviewees better understand compassion, we added “empathic understanding” as additional information in this item.

## Appendix 4 **Characteristics of study participants**

**Table A1.** **Characteristics of study participants**

| Measurement | Guangzhou n=188 | Hohhot  n=108 | P value^A^ |
| --- | --- | --- | --- |
|  |  |  |  |
| Age (years, mean/SD) | 48.2（16.9） | 51.8（17.6） | 0.08 |
| Sex (n, %) |  |  | 0.10 |
| Male | 54（28.7） | 42（38.9） |  |
| Female | 134（71.3） | 66（61.1） |  |
| Education level (n, %) |  |  | 0.58 |
| Under primary school | 21（11.2） | 8（7.4） |  |
| Middle School | 59（31.4） | 28（25.9） |  |
| High school/ Vocational school | 43（22.9） | 31（28.7） |  |
| Higher vocational school | 31（16.5） | 19（17.6） |  |
| Bachelor and graduate | 34（18.1） | 22（20.4） |  |
| Consultation order (n, %) |  |  | 0.005 |
| First time | 63（33.5） | 55（50.9） |  |
| Follow-up consultation | 125（66.5） | 53（49.1） |  |
| Visiting department (n, %) |  |  | <0.001 |
| General medicine | 131（69.7） | 81（75.0） |  |
| Internal medicine | 33（17.6） | 13（12.0） |  |
| Others | 24（12.8） | 14（13.0） |  |
| Diagnosis result (n, %) |  |  | <0.001 |
| Got a Cold | 29（15.4） | 17（15.7） |  |
| Prescribing medicine for hypertension | 21（11.2） | 16（14.8） |  |
| Prescribing medicine for diabetes | 9（4.8） | 4（3.7） |  |
| Prescribing medicine for multiple chronic diseases | 9（4.8） | 2（1.9） |  |
| Others | 120（63.8） | 69（63.9） |  |

A: P-value is for t-test continuous variables or χ2 test categorical variables.

## Appendix 5. Factor analysis to determinant the number of factors.

**Table A2. Total variance explained.**

| Factor | Initial Eigenvalues | | | Extraction Sums of Squared Loadings | | | |
| --- | --- | --- | --- | --- | --- | --- | --- |
|  | Total | % of Variance | Cumulative % | Total | % of Variance | Cumulative % | |
| 1 | 8.533 | 40.634 | 40.634 | 8.533 | 40.634 | 40.634 | |
| 2 | 1.668 | 7.943 | 48.578 | 1.668 | 7.943 | 48.578 | |
| 3 | 1.337 | 6.368 | 54.945 | 1.337 | 6.368 | 54.945 | |
| 4 | 0.978 | 4.658 | 59.603 |  |  |  | |
| 5 | 0.911 | 4.337 | 63.940 |  |  |  | |
| 6 | 0.801 | 3.813 | 67.753 |  |  |  | |
| 7 | 0.734 | 3.495 | 71.247 |  |  |  | |
| 8 | 0.610 | 2.905 | 74.153 |  |  |  | |
| 9 | 0.593 | 2.825 | 76.978 |  |  |  | |
| 10 | 0.551 | 2.622 | 79.600 |  |  |  | |
| 11 | 0.548 | 2.608 | 82.208 |  |  |  | |
| 12 | 0.510 | 2.428 | 84.636 |  |  |  | |
| 13 | 0.469 | 2.233 | 86.868 |  |  |  | |
| 14 | 0.444 | 2.113 | 88.981 |  |  |  | |
| 15 | 0.433 | 2.064 | 91.045 |  |  |  | |
| 16 | 0.389 | 1.853 | 92.897 |  |  |  | |
| 17 | 0.374 | 1.781 | 94.678 |  |  |  | |
| 18 | 0.322 | 1.535 | 96.213 |  |  |  | |
| 19 | 0.294 | 1.398 | 97.612 |  |  |  | |
| 20 | 0.264 | 1.258 | 98.869 |  |  |  | |
| 21 | 0.237 | 1.131 | 100.000 |  |  |  | |
| Extraction Method: Principal Component Analysis. | | | | | | |  |


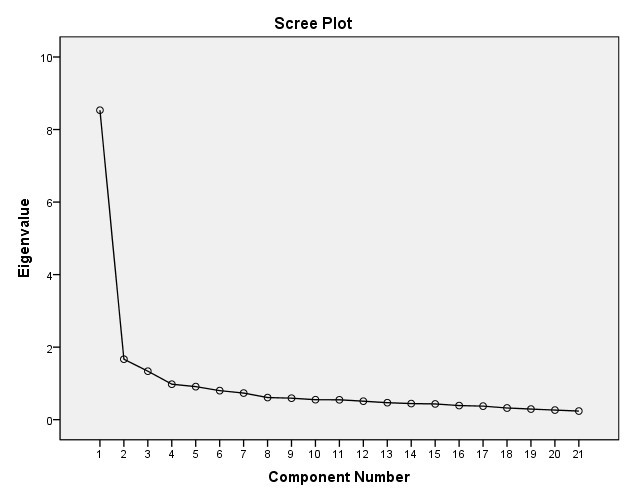


**Figure S1. Scree plot for detemination of the number of factors.**
